# Moderating role of positive aspects of caregiving in the relationship between caring burden and suicidal ideation in family caregivers of community-dwelling older adults with neurocognitive disorders

**DOI:** 10.1186/s12877-025-06147-6

**Published:** 2025-07-14

**Authors:** Zhaohua Huo, Benjamin Hon-Kei Yip, Allen Ting-Chun Lee, Sheung-Tak Cheng, Wai Chi Chan, Ada Wai-Tung Fung, Suk Ling Ma, Calvin Pak-Wing Cheng, Frank Ho-Yin Lai, Samuel Yeung-Shan Wong, Linda Chiu-Wa Lam

**Affiliations:** 1 Department of Psychiatry, Faculty of Medicine, The Chinese University of Hong Kong, Hong Kong SAR, China; 2 The Jockey Club School of Public Health and Primary Care, The Chinese University of Hong Kong, Hong Kong SAR, China; 3 Department of Health and Physical Education, The Education University of Hong Kong, Hong Kong SAR, China; 4 Department of Psychiatry, University of Hong Kong, Hong Kong SAR, China; 5 Department of Sports and Health Sciences, Hong Kong Baptist University, Hong Kong SAR, China; 6 School of Arts and Humanities, Tung Wah College, Hong Kong SAR, China; 7 Department of Social Work, Education & Community Wellbeing, Faculty of Health and Life Sciences, Northumbria University, Newcastle, UK

**Keywords:** Dementia, Cognitive impairment, Carer, Positive gain, Suicide

## Abstract

**Background:**

Psychological distress is common in family caregivers of individuals with neurocognitive disorders (NCDs). This study examined the prevalence of suicidal ideation in this population, and explored the moderating role of positive aspects of caregiving (PAC) in mitigating such severe psychological difficulties.

**Methods:**

445 older adults (NCDs: 322, normal cognition: 123) and their family caregivers were recruited from the population-based Hong Kong Mental Morbidity Survey for Older Persons. Caregivers’ suicidal ideation was screened by positive response to Item 9 of the Patient Health Questionnaire-9: thoughts of being better off dead or self-harm in the past two weeks. PAC were measured using a validated scale. A conceptual model was developed to test the mediation and moderation effects among caregiving burden, psychological distress, suicidal ideation, and PAC.

**Results:**

Nearly one in ten (9%) dementia caregivers reported suicidal ideation in the past two weeks. Higher prevalence was observed among female carers, those with comorbid mood disorders, and those caring for individuals with high comorbidity or functional dependence. Psychological distress significantly mediated the relationship between caregiving burden and suicidal ideation (80.5%, *p* = 0.023). PAC moderated this pathway: higher levels of PAC were associated with reduced suicidal ideation among carers with moderate-to-high caregiving burden (*p* < 0.05). However, a rebound in suicidal ideation was observed in caregivers experiencing both high burden and high PAC.

**Conclusions:**

Suicidal ideation among NCD caregivers is closely linked to caregiving burden and psychological distress. PAC plays a complex and non-linear moderating role in this relationship. Psychological intervention that strengthens PAC should be integrated into comprehensive caregiver support programmes, particularly for those experiencing high burden and high distress.

**Supplementary Information:**

The online version contains supplementary material available at 10.1186/s12877-025-06147-6.

## Key points

• Nearly one in ten family caregivers of older adults with dementia reports thoughts of death or suicidal ideation in Hong Kong.

• Caregiving burden significantly correlates with suicidal ideation in carers, which was mediated by psychological distress.

• Higher levels of positive experience of caregiving reduces suicidal ideation in caregivers with moderate and high caregiving burden, while a rebound in suicidal ideation was observed in those with both high burden and high PAC.

• Psychological interventions that strengthen positive experience can be integrated in carer support, especially for those with high burden and mental distress.

**Supplementary Information:**

The online version contains supplementary material available at 10.1186/s12877-025-06147-6.

## Background

Caring for older adults with neurocognitive disorders (NCDs) is challenging and burdensome for family carers. With the number of people with dementia tripling to 150 million from 2020 to 2050, and most (> 80%) being cared for at home [1, 2], a fast-growing group of family carers are foreseen. Dementia caregivers must cope with patients’ cognitive and functional deteriorations and behavioural and psychological symptoms (BPSDs), while also facing the negative impacts on their own health and wellbeing. Compared to other carers and non-carers, dementia caregivers experience heavier care workload and burden [3], more physical and psychological distress [4, 5], and lower quality and satisfaction of life [6].

Severe psychological difficulties, such as thoughts of self-harm, suicide, and homicide, are not unusual among dementia caregivers. Nearly one in three dementia caregivers express suicidal ideation, with 5.9–16.1% having attempted suicide [7]. Carers’ suicidal ideation stems from physical and emotional burnout when facing the escalating demands from their relatives [4]. High, enduring, and cumulative stress can lead to feelings of fatigue, entrapment, and hopelessness, with suicide perceived as a reprieve from them [8, 9]. Suicidal ideation is particularly pronounced when carers experience poor coping strategies, low efficacy in service seeking, and lack of family or social support [10, 11]. Depression and anxiety are also common risk factors [10, 12]. If not managed promptly, suicidal ideation may lead to suicide attempts, abusive behaviours towards care-recipients, or even homicide-suicide [13–15].

In past decades, emerging studies highlight the positive aspects of caregiving (PAC), including knowledge and skills to handle difficulties; self-accomplishment, gratification, and obligation; closer dyadic relationships, family cohesion and functionality; and personal growth, and life purpose and sharing [16, 17]. PAC can have direct, indirect or moderation effects on relieving carers’ subjective burden and psychological distress [18, 19], improving their well-being and life satisfaction [20], and deferring institutional placement for patients [21]. However, the protective or buffering roles of PAC against severe psychological distress, such as suicidal ideation, in dementia family caregivers remain unclear.

This study examined the profiles of suicidal ideation among family caregivers of persons with NCDs and explored the roles of PAC in moderating such severe psychological distress. Hypotheses include: [1] NCD staging in care-recipients correlates with levels of suicidal ideation in carers [2], psychological distress mediates the association between caregiving burden and suicidal ideation, and [3] PAC moderates the relationship between caregiving burden and suicidal ideation.

## Methods

### Study context

Hong Kong, one of the most developed economies and ageing communities globally, will face an estimate of 2.74 million older adults (36% of total population) by 2046 [22]. In 2022, nearly 693,000 older adults in Hong Kong had NCDs, with a prevalence of 21.8% in mild NCD and 9.7% in major NCD [23]. The city also faced a 15-year high suicide rate in 2022 (14.73 per 100,000), where older adults are at higher risk (44% of overall suicide cases), due to their deteriorating health and social lives, and feelings of anxiety, loneliness and abandonment in the absence of family support [24, 25].

### Study design and participants

A territory-wide, population-based and cross-sectional study, Hong Kong Mental Morbidity Survey for Older People (HKMMSOP) was conducted to from 2019 to 2022 to estimate dementia prevalence and burden in Hong Kong. A representative sample of 4,368 community-living Chinese older adults aged ≥ 60 was recruited via multi-stage random sampling. HKMMSOP used a two-phase diagnostic workflow [26]: cognitive screenings by Montreal Cognitive Assessment (MoCA) [27] and Clinical Dementia Rating (CDR) [28] for all participants in Phase 1, and clinical assessments and diagnostic workup in Phase 2 for those with positive screening results and a portion of cognitively healthy participants.

Of those attending Phase 2 assessments (positive screening: 491, normal cognition: 201), 471 family caregivers consented for in-person or phone caregiver interviews. Caregiver eligibility included: [1] aged over 18 [2], a close relative, family member, or friend providing unpaid care for an older adult aged 60 or over [3], taking major responsibility for daily communication, care, and decisions for the care-recipient, and [4] understanding and communicating in Chinese. Carers were excluded if their care-recipients were institutionalized, deceased, or if carers were physically or cognitively incapable of completing the interview. Carers caring for older adults present with normal cognitive functions but other mental disorders (e.g., mood or psychotic disorders) were also excluded, which were out of scope of this study. The final sample included 445 participant-caregiver dyads (major NCD: 67; mild NCD: 255; normal cognition: 123) (Appendix 1).

Written consent was obtained for all caregivers and care-recipients. For care-recipients with profound cognitive impairments or sensory deficits, consent and proxy information were obtained from first-degree relatives. This study followed the Helsinki declaration and was approved by the Survey and Behavioural Research Ethics Committee and Clinical Research Ethics Committee of the Chinese University of Hong Kong (Ref: 2018.494).

### Measurement

#### Demographic information

For care-recipients, data on sex, age, marital status, education level, monthly household income, and chronic conditions (Cumulative Illness Rating Scale, CIRS) [29] were collected.

For carers, data on sex, age, marital status, employment, monthly household income, caring time, familial relationship, cohabitants, number of co-carers, chronic conditions (CIRS), including presence of depression or anxiety, and quality of life (EQ-5D), were collected.

#### Clinical assessments

For care-recipients, NCD diagnosis and subtype (Alzheimer’s, vascular, mixed, other) were determined using Diagnostic and Statistical Manual of Mental Disorders-5 (DSM-5) criteria. Dementia stage was determined by CDR, with scores of 1, 2 and 3 indicating mild, moderate and severe dementia [28]. Mild NCD and mild cognitive impairment (MCI), as well as major NCD and dementia, were used interchangeably. Other clinical assessments of care-recipients included MoCA, activity function (Disability Assessment for Dementia, DAD) and neuropsychiatric symptoms (Neuropsychiatric Inventory Questionnaire, NPI-Q) [30, 31].

#### Caregiving burden

Caregiving burden was measured by the Zarit Burden Interview (ZBI), a 22-item scale assessing carers’ perceptions of their health, well-being, finances, social life, and relationship with care-recipients [32, 33]. Each ZBI item was rated on a 5-point Likert scale from 0 (not at all) to 4 (nearly always), with total scores ranging from 0 to 88 (none to mild: 0–20; mild to moderate: 21–40; moderate to severe: 41–60; severe: 61–88).

#### Psychological distress

Carers’ psychological distress was measured by the 2-item module of Patient Health Questionnaire (PHQ-2). Each PHQ-2 item was rated on a 5-point Likert scale from 0 (not at all) to 3 (nearly every day), with scores of 3 or greater indicating high risk of major depressive disorder [34].

#### Suicidal ideation

Suicidal ideation was assessed by the single item from Patient Health Questionnaire-9 (PHQ-9): “Over the past two weeks, how often have you had thoughts that you would be better off dead, or thoughts of hurting yourself in some way?” [35]. The item was rated on a 5-point Likert scale from 0 (not at all) to 3 (nearly every day), with higher scores indicating higher intensity of suicidal thoughts.

#### Positive aspects of caregiving

Positive caregiving experience was measured by the Positive Aspects of Caregiving Scale (PACS), which consists of two constructs: self-affirmation (confidence and capability perceived by caregivers) and outlook on life (enhanced interpersonal relationships and positive life orientation) [36, 37]. Each PACS item was rated on a 5-point Likert scale from 1 (disagree a lot) to 5 (agree a lot), with total scores ranging from 9 to 45 (low: 9–27; moderate: 28–36, and high: 37–45).

### Statistical analyses

No missing data was found on caregiver outcomes. Missing data (< 2%) on care-recipients were imputed using informant reports (for demographics) or mean values of the same participant group (for CIRS, MoCA, DAD). Sample weighting adjusted for oversampling and non-response in the caregiver survey.

Demographic and outcome differences across three cognitive status groups (normal cognition, mild NCD, major NCD) were examined by Chi-square tests, ANOVA, and post-hoc t-tests. A conceptual framework grounded in existing theories and evidence was developed to illustrate the mediating role of psychological distress in the relationship between caregiver burden and suicidal ideation, as well as the moderating effect of PAC (Appendix 2). Hierarchical regression models were employed to examine the relationships among these variables within the proposed framework.

In step one, generalized linear regression models were built on three caregiver outcomes (caring burden, psychological distress, suicidal ideation). A stepwise selection strategy (ruling in: *P* < 0.10, ruling out: *P* > 0.20) was employed to identify significant covariates, ensuring model parsimony and minimising multicollinearity (variance inflation factors remaining < 5). In step two, mediation analyses were conducted to assess whether psychological distress mediated the relationship between caregiving burden and suicidal ideation, controlling for confounders identified in step one. In step three, the moderation role of PAC was further examined within the mediation pathway. Specifically, we tested whether PAC influenced the strength of the indirect effect of caregiving burden on suicidal ideation via psychological distress, again adjusting for relevant covariates. Simple slope analyses were used to illustrate how varying levels of PAC modified the relationship between caring burden and suicidal ideation [38].

Sensitivity analysis was conducted firstly by restricting the sample to NCD carers only, and secondly by analysing the separate effects of two constructs of PAC (self-affirmation and outlook on life). The significance level was set at 0.05, except in Bonferroni corrections at 0.0167. All analyses were completed using IBM SPSS software 21.0.

## Results

### Characteristics of participants

A total of 445 pairs of older adults and carers were included. Care-recipients with NCDs, compared to normal controls, were predominantly female (> 50%), older (mean age > 70), had lower education and income levels, non-married (> 30%), not working (> 90%), and had lower cognitive and physical functions (Appendix 3). On average, care-recipients lived with around 2 cohabitants, while nearly one in ten participants lived alone.

Carers in the three cognitive status groups were comparable in sex (> 60% female), employment status (> 60% not working), household income, living with care-recipients (> 70%), and comorbidity score. However, carers in NCD groups were younger (major NCD: 60.8; mild NCD: 59.1), more often non-spouses (major NCD: 78.7%; mild NCD: 47.3%), caring for longer hours, more assisted by co-carers, and reporting lower quality of life (Appendix 3). Unadjusted correlations between caregiver outcomes and different covariates are shown in Appendix 4.

### Caregiver outcomes and associated factors

Caring burden: ZBI scores were significantly higher among NCD carers (major NCD: 21.1 ± 15.3; mild NCD: 7.5 ± 8.1), so was the rate of moderate-to-severe burden (major NCD: 45.7%; mild NCD: 7.5%) (Table 1). Factors associated with higher caring burden included severer NCD stage, higher income levels, more comorbidities and neuropsychiatric symptoms in care-recipients, and being female, non-spouses, no cohabiting child, longer caring hours, and mood disorders in carers (Table 2).

**Table 1:** Caregiver outcomes by cognitive status groups

Frequency (%)/Mean ± SD	Diagnostic group			
	Group A: Normal control (*n* = 132)	Group B: Mild NCD (MCI) (*n* = 264)	Group C: Major NCD (Dementia) (*n* = 68)	Differences^a^ (*P*-value)
**Caring burden**				
ZBI score	3.5 ± 5.4	7.5 ± 8.1	21.1 ± 15.3	< 0.001 (a < b < c)
ZBI severity: None to mild (ZBI: 0–20)	121 (98.4%)	236 (92.5%)	37 (55.2%)	< 0.001
Mild to moderate (ZBI: 21–40)	2 (1.6%)	17 (6.7%)	21 (31.3%)	
Moderate to severe (ZBI: 41–88)	0 (0.0%)	2 (0.8%)	9 (13.4%)	
**Psychological distress**				
PHQ-2 score	0.7 ± 1.2	0.9 ± 1.3	1.4 ± 1.8	0.002 (a, b < c)
Major depressive disorder: positive (PHQ-2: 3–6)	10 (8.1%)	30 (11.8%)	9 (13.4%)	0.451
**Suicidal ideation**				
Item score (0–3)	0.06 ± 0.28	0.05 ± 0.31	0.14 ± 0.46	0.151 (n.s.)
Frequency: not at all	117 (95.9%)	245 (96.5%)	61 (91.0%)	0.108
Several days	4 (3.3%)	7 (2.8%)	3 (4.5%)	
Half of days/everyday	1 (0.8%)	2 (0.8%)	3 (4.5%)	
**Positive aspects of caregiving**				
PACS: total score	28.9 ± 3.8	30.5 ± 5.0	33.0 ± 6.2	< 0.001 (a < b < c)
PACS: self-affirmation score	19.5 ± 2.7	20.5 ± 3.5	22.3 ± 4.1	< 0.001 (a < b < c)
PACS: outlook on life score	9.5 ± 1.3	10.0 ± 1.9	10.7 ± 2.5	< 0.001 (a < b < c)
PACS level: Low (PACS: 9–27)	78 (63.4%)	117 (45.9%)	16 (23.9%)	< 0.001
Moderate (PACS: 28–36)	37 (30.1%)	106 (41.6%)	32 (47.8%)	
High (PACS: 37–45)	8 (6.5%)	32 (12.5%)	19 (28.4%)	

^a^Differences between subgroups were examined by chi-square for categorical variables and ANOVA with post-hoc t-tests for continuous variables

*NCD *Neurocognitive disorder, *n.s *not significant, *PACS *Positive aspects of Caregiving scale, *PHQ-2 *Patient Health Questionnaire-2, *SD *Standard deviation, *ZBI *Zarit Burden Inventory

**Table 2:** Associated factors of caregiver outcomes based on Stepwise regression models

Final model fit	ZBI	PHQ-2	Suicidal ideation	PACS
Adjusted R-square (P-value)	R^2^ = 0.519^***^	R^2^ = 0.209^***^	R^2^ = 0.127^***^	R^2^ = 0.128^***^
**Care-recipients**	Coefficient: β (SE)			
Diagnosis: Mild NCD (ref: Normal)	1.656 (0.852)^	(Not included)	(Not included)	(Not included)
Major NCD	7.065 (1.375)^***^	(Not included)	(Not included)	(Not included)
Sex: Female	(Not included)	−0.345 (0.118)^**^	(Not included)	(Not included)
Age	(Not included)	(Not included)	(Not included)	0.099 (0.031)^**^
Monthly income: ≥HK$15,000 (ref:< HK$5,999)	2.094 (0.762)^**^	(Not included)	(Not included)	(Not included)
Cohabitants: ≥2 (ref: living alone)	(Not included)	0.305 (0.129)^*^	(Not included)	(Not included)
Comorbidity score (CIRS)	0.400 (0.146)^**^	0.121 (0.032)^***^	0.030 (0.008)^***^	(Not included)
Cognitive function (MoCA)	(Not included)	(Not included)	(Not included)	−0.092 (0.051)^
Physical function (DAD)	(Not included)	(Not included)	−0.004 (0.001)^***^	−0.037 (0.016)^*^
Neuropsychiatric symptoms (NPI)	0.243 (0.040)^***^	0.026 (0.006)^***^	(Not included)	(Not included)
**Informal caregivers**	Coefficient: β (SE)			
Sex: Female	1.848 (0.752)^*^	(Not included)	0.064 (0.031)^*^	(Not included)
Relationship: Spouse	−4.155 (0.752)^***^	(Not included)	(Not included)	(Not included)
Number of cohabitating children	−1.698 (0.498)^**^	−0.179 (0.088)^*^	(Not included)	(Not included)
Monthly caring hours	0.052 (0.005)^***^	0.003 (0.001)^***^	(Not included)	(Not included)
Comorbidity of depression/anxiety	3.307 (1.603)^*^	1.629 (0.271)^***^	0.247 (0.069)^***^	(Not included)

^a^Final model fit was determined by generalized linear regression and stepwise method (ruling in: *P* < 0.10, ruling out: *P* > 0.20) to pick out most significant covariates (^^^, *P* < 0.10; ^*^, *P* < 0.05; ^**^, *P* < 0.01; ^***^, *P* < 0.001)

*CIRS *Cumulative Illness Rating Scale, *DAD *Disability Assessment for Dementia, *MoCA *Montreal Cognitive Assessment, *NCD N*eurocognitive disorder, *NPI *Neuropsychiatric Inventory, *PACS *Positive aspects of Caregiving scale, *PHQ-2 *Patient Health Questionnaire-2, *ZBI *Zarit Burden Inventory

Psychological distress: PHQ-2 scores were higher in major NCD (1.4 ± 1.8) and mild NCD (0.9 ± 1.3) carers (Table 1), also linked to care-recipients demographics (female, cohabitant ≥ 2, comorbid conditions and neuropsychiatric symptoms) and caregiver factors (no cohabiting children, longer caring hours, and mood disorders).

Suicidal ideation: Two-week occurrence and intensity of suicidal ideation were higher in carers of major NCD (9% or 0.14 ± 0.46) compared to mild NCD (3.6%, 0.05 ± 0.31) and normal control (4.1%, 0.06 ± 0.28) (Table 1). Suicidal ideation was significantly associated with carers’ sex and mood disorders, and care-recipients’ chronic conditions and physical functioning (Table 2).

Positive experience: PACS scores were higher in carers of major NCD (33.0 ± 6.2) and mild NCD (30.5 ± 5.0), so were subdimensions on self-affirmation and outlook on life (Table 1). Higher PACS scores were found in carers of care-recipients with older age and lower cognitive and physical functions (Table 2).

### Mediation effect of psychological distress on burden and suicidal ideation

Adjusted analysis showed caregiving burden was positively correlated with psychological distress (*r* = 0.262, *P* < 0.001) and suicidal ideation (*r* = 0.130, *P* < 0.001), while psychological distress was correlated with suicidal ideation (*r* = 0.422, *P* < 0.001) (Appendix 4). Mediation analysis confirmed psychological distress mediated the relationship between caring burden and suicidal ideation (total effect: 100%, β = 0.005, *P* = 0.024; direct effect: 19.5%, β = 0.001, *P* = 0.643; indirect effect: 80.5%, β = 0.004, *P* = 0.007) (Appendix 5).

### Moderation effect of PAC on suicidal ideation

**Fig. 1:**
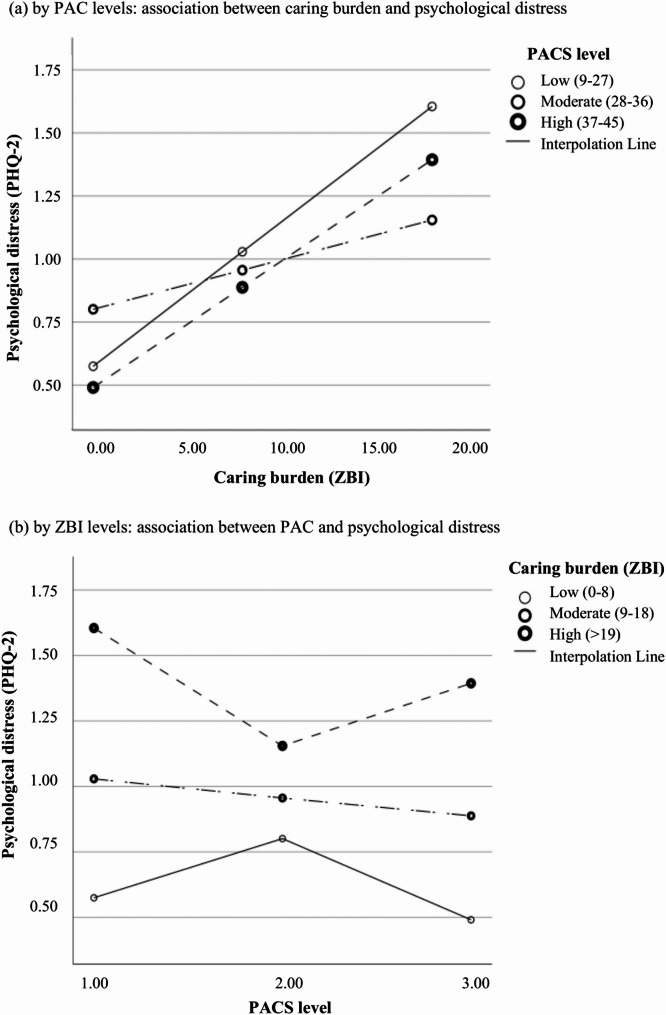
Moderation effects of PAC levels on caring burden and psychological distress. **a** by PAC levels: association between caring burden and psychological distress. **b** by ZBI levels: association between PAC and psychological distress

**Fig. 2:**
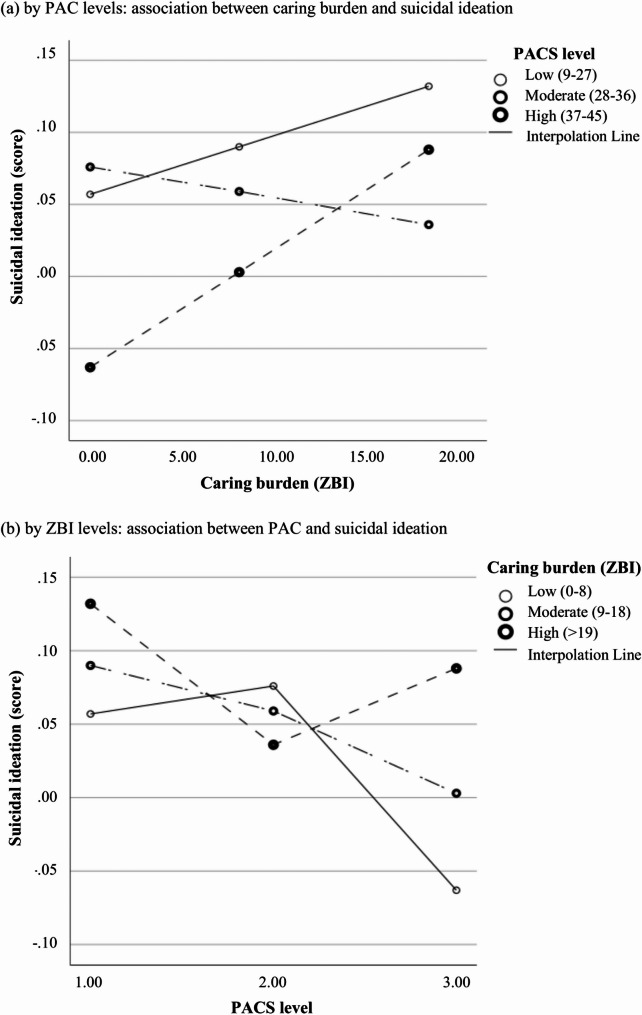
Moderation effects of PAC levels on caring burden and suicidal ideation. **a** by PAC levels: association between caring burden and suicidal ideation. **b** by ZBI levels: association between PAC and suicidal ideation

Adjusted correlation showed no significant associations between PACS and the three carer outcomes (*r*=−0.050-0.042, *P* > 0.05) (Appendix 4). In contrast, moderation effects were detected of PACS in the pathways between caring burden, psychological distress, and suicidal ideation (Table 3). The relationship between caring burden and psychological distress was buffered when carers had a moderate level of PAC (*P* = 0.015), so was the relationship between caring burden and suicidal ideation (*P* = 0.022) (Figs. 1 and 2). Similarly, higher levels of PAC were associated with fewer depressive symptoms and suicidal ideation when carers were suffering moderate to high burden (Figs. 1 and 2). Sensitivity analyses showed consistent findings (not shown).

**Table 3:** Moderation effects of PAC between caring burden, psychological distress and suicidal ideation

Modelling: X*PACS -> Y^a^	Model fit	Effect of X: β (SE), *P*-value
**Step 1. Path estimates**		
ZBI*PACS -> PHQ-2	R^2^ = 0.227^***^	Interaction: F = 4.223, *P* = 0.015
Low PACS		0.057 (0.011), *P* < 0.001
Moderate PACS		0.020 (0.010), *P* = 0.053
High PACS		0.050 (0.018), *P* = 0.005
ZBI*PACS -> SI	R^2^ = 0.230^***^	Interaction: F = 3.840, *P* = 0.022
Low PACS		0.004 (0.003), *P* = 0.143
Moderate PACS		−0.002 (0.002), *P* = 0.370
High PACS		0.008 (0.005), *P* = 0.070
PHQ-2*PACS -> SI		Interaction: F = 0.959, *P* = 0.384
Low PACS		0.090 (0.017), *P* < 0.001
Moderate PACS		0.109 (0.018), *P* < 0.001
High PACS		0.060 (0.032), *P* = 0.065
**Step 2. Mediation estimates**		Effect of X on Y: β (SE)
Direct effect		
Low PACS		0.004 (0.003), *P* = 0.143
Moderate PACS		−0.002 (0.002), *P* = 0.370
High PACS		0.008 (0.005), *P* = 0.070
Indirect effect		
Low PACS		0.005 (0.003), *P* = 0.025
Moderate PACS		0.002 (0.002), *P* = 0.095
High PACS		0.003 (0.003), *P* = 0.189

^a^Moderation roles of PAC were examined in the three paths: [1] ZBI*PACS -> PHQ-2 [2], PHQ-2*PAC -> SI, and [3] ZBI*PAC -> SI in the mediation model (ZBI -> PHQ-2 -> SI). Simple slope analysis was performed to reveal differences in the relationship between independent (X) and dependent (Y) variables across moderator (PACS) scoring at low (−1 SD), medium (mean) and high (+ 1SD) levels

*PACS *Positive Aspects of Caregiving scale, *PHQ-2 *Patient Health Questionnaire-2, *SD *Standard deviation, *SI *Suicidal ideation, *ZBI *Zarit Burden Inventory

## Discussion

### Main findings

#### Suicidal ideation in dementia caregivers and associated factors

Our survey in Hong Kong found 9% of family carers of people with dementia in the community reported suicidal ideation in the past two weeks, twice the rate observed among non-dementia caregivers. This prevalence was comparable to Japan (10.6%) [39], highlighting the significant mental health challenges faced by dementia caregivers in East Asian contexts. However, the rate of suicidal ideation in dementia caregivers in Hong Kong is somehow lower than estimates reported in a systematic review of eight studies (32%), as well as individual studies from South Korea (17–46%) [40], Australia (16–26%) [12, 14], Canada (52% among carers aged 55 or over) [15]. These discrepancies may be attributed to the methodological heterogeneity across studies in sampling strategies, measurement tools, and recall periods [7, 11, 40]. Notably, population-based studies often report lower prevalence rates (e.g., an Australian study: 2.4% [14]). Besides dementia caregivers, suicidal ideation was also prevalent in caregivers of persons with other long-term illnesses and disabilities, such as cancer (a meta-analysis on eleven studies: 11% [41]; Korea: 18% [42]) and mental disorders (Brazil: 12.5% [43]; Turkey: 17.9% [44]). These findings underscore that suicidal ideation is a widespread concern across caregiving contexts, regardless of disease type.

Several factors were identified as significantly associated with suicidal ideation among caregivers, including caring for older adults with severe cognitive impairment and multiple chronic conditions, as well as caregiver characteristics such as being female or having a mood disorder. Greater dementia severity, often indicative of increased patient dependence and caregiver burnout, has been linked to elevated suicide risk among caregivers [40]. We also echo previous studies that female carers tend to experience greater responsibilities and psychological stress compared to their male counterparts [45, 46]. This disparity may be further exacerbated by cultural expectations surrounding filial obligation and traditional gender roles in Chinese society, which often discourage women from seeking or receiving external support [47]. Moreover, the presence of mood disorder, such as anxiety or depression, has been repeatedly identified as a prominent risk factor for suicidal ideation in caregivers [11, 14, 41, 42]. These conditions may interact with the chronic stress of caregiving, compounding psychological vulnerability. Depression in caregivers may not only precede caregiving but can also emerge as a consequence of it, shaped by the complex interaction of caregiver characteristics, care-recipient demands, and sociocultural context [48].

In unadjusted correlations, factors such as unemployment, extended caregiving hours, and the presence of chronic conditions among caregivers were associated with increased suicidal ideation. These findings reflect the cumulative impact of physical, psychological, and financial strain, which may diminish caregivers’ capacity to sustain care for both their relatives and themselves [8, 41, 49].

#### Psychological distress as a mediation between caring burden and suicidal ideation

Psychological distress was found to significantly mediate the relationship between caregiving burden and suicidal ideation, aligning with findings from previous studies [14]. In our sample, female and non-spouse caregivers reported higher levels of caregiving burden, while those caring for male older adults experienced greater psychological distress. Contrary to earlier research suggesting that spousal caregivers tend to report higher levels of distress [41, 49], our findings indicate that non-spouse caregivers (e.g., adult children/children in-law) within the Chinese population also experience substantial caregiving burden. Compared to spouse caregivers, non-spouse carers in our study were younger (mean age: 49.8 vs. 69.0), more likely to be employed (64% vs. 18%), less likely to cohabit with the care recipient (47% vs. 99%), and more likely to hire a domestic helper for caregiving support (21% vs. 6%). Nearly half of the non-spouse caregivers also had a child living with them. These demographic and contextual differences suggest that non-spouse caregivers often juggle multiple family and social roles, contributing to higher scores on specific items of the ZBI, including (1) imbalance of responsibilities between caring, family and work (2), lack of personal time, privacy, and social life (3), perceived high dependence of care-recipient, and (4) inadequate preparedness and capacity to provide care. These findings underscore the multifaceted challenges faced by non-spouse caregivers and highlight the need for tailored support strategies that address their unique burdens and responsibilities in caring for relatives with NCDs.

Larger household sizes, measured by the number of cohabitants living with the care-recipient, were also associated with higher psychological distress among caregivers in our study. This may reflect the compounded demands of simultaneously addressing the diverse needs of multiple family members, such as caring for two elderly relatives, a spouse, or underage children, while also navigating strained living conditions and complex family dynamics [9]. Conversely, the presence of more children cohabiting with the caregiver was linked to lower levels of caregiving burden and psychological distress. This suggests that support from children, particularly in the case of older caregivers, may play a protective role by sharing caregiving responsibilities and providing emotional or practical assistance. These findings underscore the dual nature of family structure in caregiving - where larger households can either intensify stress or serve as a source of support, depending on the roles and relationships within the family unit.

#### Moderation roles of PAC in relieving psychological distress and suicidal ideation

While previous studies have explored the direct effects of positive aspects of caregiving (PAC) on caregivers’ psychological health, subjective well-being, and coping strategies [37, 50], relatively few have examined its buffering role in mitigating the negative consequences of caregiving burden on psychological distress. Walker et al. (2016) found that among female caregivers, PAC moderated the relationship between caregiving burden and depression, and specifically, high levels of PAC were associated with lower depression only in those experiencing low, but not high, caregiving burden [51]. Our findings echo this non-linear pattern. We observed that moderate levels of PAC were most effective in buffering the negative impact of caregiving burden on psychological outcomes. It was also reflected as while higher PAC levels were generally associated with reduced psychological distress and suicidal ideation compared to low PAC, a rebound in negative outcomes was noted among caregivers experiencing both high PAC and high caregiving burden. This may reflect the complex and parallel processes of positive and negative caregiving experiences in caregiving, which could be shaped by shared factors (e.g., care recipient’s cognitive and functional status, caregiving hours) and distinct caregiver characteristics [52–54]. Among caregivers with mild to moderate burden, greater PAC was linked to enhanced feelings of accomplishment, improved relationship quality, better coping strategies, and higher life satisfaction - factors that help regulate and counterbalance the emotional toll of caregiving [55–57].

However, under conditions of high burden, caregivers often face compounded stressors, including severe care-recipient symptoms, personal physical and mental health challenges, limited time and privacy, and competing family and social responsibilities [51]. In such cases, even a strong sense of caregiving purpose or self-affirmation may not translate into sufficient resilience or coping capacity for caregivers to manage their excessively physical and emotional fatigue [8]. Some caregivers have even reported suicidal thoughts following the death or institutionalization of the care recipient, expressing a loss of purpose and emotional exhaustion [8]. For these high-burden caregivers, interventions may focus more on effective ways to reduce caregiving demands, such as through formal care services or shared responsibilities with other family members, rather than solely enhancing PAC [51].

Finally, the moderating role of may vary by caregiver gender and cultural context. Wong et al. (2019) found that PAC had a stronger buffering effect on depression among Chinese wife caregivers compared to husbands [46]. Cultural values, such as filial obligation, family hierarchy, intergenerational expectations, and patrilocal norms, can shape both the caregiving experience and the perception of PAC, as well as influence how caregivers interpret disease symptoms and caregiving roles [51, 58, 59]. These findings, along with our own, highlight the need for further qualitative and quantitative research into the contextual formation of PAC and its mechanisms in influencing caregiver psychological outcomes, particularly among Chinese dementia caregivers facing high caregiving burden [51, 58]. In our sample, caregivers with both high PAC and high psychological distress were typically young-old women (aged 60–74), unemployed, and cohabiting with and caring for their old-old parents or parents-in-law. As the population ages, a new “sandwich” generation is emerging, which has to simultaneously manage the care of elderly relatives, frail spouses, and dependent children, while navigating retirement-related income loss, increased financial strain, and conflicts between personal needs (leisure time) and caregiving responsibilities. Their lived experiences are essential to develop targeted, culturally sensitive support strategies.

### Limitations

Firstly, this study is exploratory in nature, aiming to examine the pathway from caregiving burden to suicidal ideation among dementia caregivers, and the moderating role of PAC within this relationship. While our analytical models are built on prior theories and evidence, the cross-sectional design alone limits our ability to assess temporal dynamics, infer longitudinal associations, or establish causal relationships between variables [60]. Similarly, while we included caregivers with mood disorders, which were significantly associated with suicidal ideation, the cross-sectional data do not allow us to determine whether these psychological symptoms were a consequence of caregiving or pre-existing conditions.

Secondly, despite employing a population-based sampling strategy and applying sample weighting, some degree of sampling bias is unavoidable. Older adults with severe cognitive or functional impairments, as well as individuals from hard-to-reach populations, were less likely to participate. Additionally, caregivers experiencing overwhelming caregiving demands may have been unable to take part in the survey, potentially leading to an underestimation of suicidal ideation prevalence.

Lastly, while the item 9 of PHQ-9, “thoughts that you would be better off dead, or thoughts of hurting yourself in some way”, was widely used as a proxy to measure suicidal ideation, it does not fully capture the complexity of suicidal thoughts. Specifically, the phrasing of “thoughts that you would be better off dead” in the assessment item may conflate passive death wishes with active suicidal thoughts and intent, and its two-week recall period may underestimate the true prevalence of suicidal ideation among caregivers. This subtle yet critical distinction based on the single item may misestimate the prevalence and severity of suicidal ideation in caregivers, further limiting accurate risk stratification and the development of targeted interventions [61]. Therefore, future studies should consider using more comprehensive and validated instruments, such as Suicidal Ideation Questionnaire or Beck Scale for Suicide Ideation, to differentiate between passive death wishes and active suicidal thoughts, as well as to assess suicidal planning and attempts [62].

### Implications

Given the notably high prevalence of suicidal ideation among dementia family carers, timely risk assessment and early detection are critical for effective prevention and intervention. Health professionals should be adequately trained to screen for suicidal ideation and behaviours in caregivers as part of routine assessments [63]. Particular attention should be paid to high-risk groups, including female caregivers, those caring for individuals with severe NCDs, and caregivers experiencing high levels of burden or comorbid mental health conditions [10, 15, 42]. Although research on suicide-specific interventions for caregivers remains limited, emerging strategies, such as psychosocial support groups and mentalizing imagery therapy, have shown promise in addressing suicidal ideation in caregivers, and can be integrated into broader caregiver support systems [64].

Given the mediating role of psychological distress and the moderating effect of PAC, positive caregiving experiences can serve as protective factors to prevent and manage psychological distress and suicidal ideation. Particularly among carers with moderate or high caring burden, higher levels of PAC were associated with reduced psychological distress and suicidal ideation. This suggests that interventions aimed at enhancing PAC may be effective in promoting caregiver well-being, such as benefit-finding, positive reappraisal, group-based positive psychology, occupation-based interventions, acceptance and commitment therapy, positive mood therapy, and active life therapy [65–68]. These approaches can be culturally adapted and embedded within comprehensive caregiver support networks [51].

The non-linear moderating role of PAC must be acknowledged, given a rebound in psychological distress among caregivers with both high PAC and high caregiving burden. This suggests that for these individuals, while fostering positive experiences is beneficial, more comprehensive support is indispensable, including access to formal and respite care services to alleviate physical and emotional burden, opportunities to build social networks, strategies to enhance coping and personal growth, and adequate financial and workplace support [69].

Finally, longitudinal and experimental studies are needed to establish causal relationships and capture the dynamic interplay between caregiving burden, PAC, and psychological outcomes over time [70]. Future qualitative and quantitative research should also explore the contextual formation and interpretation of PAC, particularly among the emerging “sandwich” generation of Chinese dementia caregivers identified in our study [51]. Investigating their lived experiences contributing to suicidal ideation, including family conflict, coping and placement challenges, and perceptions of familism, exhaustion, stigma, injustice, and loneliness, will be essential for developing context-tailored and culture-sensitive interventions [9, 12, 71, 72].

## Conclusions

Suicidal ideation was prevalent in nearly one in ten dementia family caregivers, with the highest rates observed among female carers, those comorbid with mood disorders, and those caring for older persons with multiple chronic conditions and significant functional impairment. Given the moderating role of PAC in alleviating suicidal ideation in caregivers experiencing moderate-to-high caring burden, strategies to foster and enhance PAC should be integrated into comprehensive caregiver support services.

## Supplementary Information

Supplementary Material 1: Appendix 1. Recruitment of participants. Appendix 2. Conceptual model on caregiving burden, psychological distress, positive experience and suicidal ideation. Appendix 3. Demographics of older participants and informal caregivers. Appendix 4. Bivariate correlations between demographics and caregiver outcomes, before and after adjustment. Appendix 5. Mediation analysis on caring burden, psychological distress and suicidal ideation

## Data Availability

Study data, analytic methods, or materials are available from the Principal Investigators of each site upon reasonable request. The study was not preregistered before.

## Abbreviations

BPSD: Behavioural and Psychological Symptom of Dementia
CDR: Clinical Dementia Rating
CIRS: Cumulative Illness Rating Scale
DSM-5: Diagnostic and Statistical Manual of Mental Disorders-5
DAD: Disability Assessment for Dementia
HKMMSOP: Hong Kong Mental Morbidity Survey for Older People
MCI: Mild Cognitive Impairment
MoCA: Montreal Cognitive Assessment
NCD: Neurocognitive Disorder
NPI-Q: Neuropsychiatric Inventory Questionnaire
PHQ-2: Patient Health Questionnaire-2
PHQ-9: Patient Health Questionnaire-9
PAC: Positive aspects of caregiver
PACS: Positive Aspects of Caregiving Scale
ZBI: Zarit Burden Interview
