# Supplementary material for: Moderating role of positive aspects of caregiving in the relationship between caring burden and suicidal ideation in family caregivers of community-dwelling older adults with neurocognitive disorders
Source: BMC Geriatr. 2025 Jul 14;25:524. doi: 10.1186/s12877-025-06147-6 (PMC12257795; doi:10.1186/s12877-025-06147-6)
Supplement: Supplementary file 1 — Supplementary Material 1: Appendix 1. Recruitment of participants. Appendix 2. Conceptual model on caregiving burden, psychological distress, positive experience and suicidal ideation. Appendix 3. Demographics of older participants and informal caregivers. Appendix 4. Bivariate correlations between demographics and caregiver outcomes, before and after adjustment. Appendix 5. Mediation analysis on caring burden, psychological distress and suicidal ideation [file 12877_2025_6147_MOESM1_ESM.docx]

**Article title**: Moderating role of positive aspects of caregiving in the relationship between caring burden and suicidal ideation in family caregivers of community-dwelling older adults with neurocognitive disorders

**Author names**: Zhaohua Huo, Benjamin Hon-Kei Yip, Allen Ting-Chun Lee, Sheung Tak Cheng, Wai Chi Chan, Ada Wai-Tung Fung, Suk Ling Ma, Calvin Pak-Wing Cheng, Frank Ho-Yin Lai, Samuel Yeung-Shan Wong, Linda Chiu-Wa Lam

**Corresponding author:**

Linda Chiu-Wa Lam

Department of Psychiatry, Faculty of Medicine, The Chinese University of Hong Kong

Email: cwlam@cuhk.edu.hk

**Appendices**

Contents

[Appendix 1. Recruitment of participants 3](#_Toc198595944)

[Appendix 2. Conceptual model on caregiving burden, psychological distress, positive experience and suicidal ideation 4](#_Toc198595945)

[Appendix 3. Demographics of older participants and informal caregivers 5](#_Toc198595946)

[Appendix 4. Bivariate correlations between demographics and caregiver outcomes, before and after adjustment 6](#_Toc198595947)

[Appendix 5. Mediation analysis on caring burden, psychological distress and suicidal ideation 7](#_Toc198595948)

# Appendix 1. Recruitment of participants

Eligible households

(n=4,810)

Not complete

(n=442)

Phase 2 assessment (n=692)

- Screened positive (491)

- Screened negative (201)

Invited for Phase 2 (n=1,398)

- Screened positive (1,148)

- Screened negative (250)

Not complete: refusal, no response, no time, physical inability (n=706)

Phase 1 assessment (n=4,368)

- Screened positive (1,148)

- Screened negative (3,220)

Caregiver interview

(n=471)

Included in analysis

(n=445; normal: 123, mild NCD: 255, major NCD: 67)

Excluded: normal cognition with other mental disorders

(n=26)

Not complete: refusal, no response, hospitalization, and no carer identified (n=204)

# Appendix 2. Conceptual model on caregiving burden, psychological distress, positive experience and suicidal ideation

Psychological distress (PHQ-2)

Caregiving burden (ZBI)

Suicidal ideation

Positive aspects of caregiver

# Appendix 3. Demographics of older participants and informal caregivers

| Frequency (%) / Mean±SD | **Cognitive status group** | | | |
| --- | --- | --- | --- | --- |
|  | Group A:  Normal control (n=123) | Group B: Mild NCD (MCI) (n=255) | Group C: Major NCD (Dementia) (n=67) | Differences^a^  (P-value) |
| **Care-recipients** |  |  |  |  |
| Gender: Female | 55 (44.7%) | 140 (54.9%) | 44 (65.7%) | 0.018 |
| Age | 68.3±6.9 | 73.6±7.8 | 84.3±8.8 | <0.001 (a<b<c) |
| Education level: Primary school or below | 40 (32.8%) | 153 (60.0%) | 50 (74.6%) | <0.001 |
| Middle school | 63 (51.6%) | 83 (32.5%) | 14 (20.9%) |  |
| College | 19 (15.6%) | 19 (7.5%) | 3 (4.5%) |  |
| Marital: Married/Cohabited | 102 (82.3%) | 173 (67.8%) | 33 (49.3%) | <0.001 |
| Widowed | 13 (10.5%) | 59 (23.1%) | 30 (44.8%) |  |
| Divorced/Separate/Single | 9 (7.3%) | 23 (9.0%) | 4 (6.0%) |  |
| Employment: Working | 28 (22.8%) | 25 (9.8%) | 3 (4.5%) | <0.001 |
| Monthly household income: HK$5,999 or lower | 24 (19.5%) | 59 (23.1%) | 30 (44.8%) | <0.001 |
| HK$6,000-14,999 | 42 (34.1%) | 111 (43.5%) | 19 (28.4%) |  |
| HK$15,000 or higher | 57 (46.3%) | 85 (33.3%) | 18 (26.9%) |  |
| Living alone: Yes | 14 (11.4%) | 28 (11.0%) | 8 (11.9%) | 0.974 |
| Number of cohabitants | 2.6±1.3 | 2.8±1.4 | 2.9±1.2 | 0.446 (n.s.) |
| Comorbidity score (CIRS) | 3.3±2.1 | 4.4±2.4 | 5.6±3.6 | <0.001 (a<b<c) |
| Cognitive function (MoCA) | 24.0±4.1 | 19.5±4.3 | 10.1±6.0 | <0.001 (a>b>c) |
| Physical function (DAD) | 99.4±1.5 | 96.9±5.5 | 66.7±34.8 | <0.001 (a, b>c) |
| Neuropsychiatric symptoms (NPI) | 2.8±4.0 | 5.4±9.2 | 10.1±14.0 | <0.001 (a<b<c) |
| Type of NCD: Alzheimer’s disease | / | 56 (22.0%) | 11 (16.4%) | 0.320 |
| **Family caregivers** |  |  |  |  |
| Gender: Female | 82 (66.7%) | 152 (59.6%) | 46 (68.7%) | 0.236 |
| Age | 63.4±10.6 | 59.1±15.2 | 60.8±14.3 | 0.018 (a>b) |
| Employment: Working | 41 (33.3%) | 104 (40.8%) | 25 (37.3%) | 0.372 |
| Monthly household income: HK$9,999 or lower | 40 (32.5%) | 75 (29.4%) | 24 (35.8%) | 0.620 |
| HK$10,000-29,999 | 52 (42.3%) | 104 (40.8%) | 22 (32.8%) |  |
| HK$30,000 or higher | 31 (25.2%) | 76 (29.8%) | 21 (31.3%) |  |
| Relationship: Spouse | 95 (77.2%) | 133 (52.2%) | 21 (31.3%) | <0.001 |
| Cohabiting with care-recipient: Yes | 103 (83.7%) | 186 (72.9%) | 50 (74.6%) | 0.066 |
| Cohabiting with any child: Yes | 55 (44.7%) | 123 (48.2%) | 23 (34.3%) | 0.125 |
| Monthly caring hours | 26.5±35.3 | 40.0±53.9 | 144.5±151.8 | <0.001 (a, b<c) |
| Number of co-caregivers | 1.0±1.0 | 1.3±1.1 | 1.5±1.0 | 0.004 (a<b,c) |
| Comorbidity score (CIRS) | 2.6±1.7 | 2.4±1.9 | 2.1±1.8 | 0.285 (n.s.) |
| Comorbidity of depression/ anxiety | 5 (4.1%) | 13 (5.1%) | 6 (9.0%) | 0.334 |
| Quality of life (EQ-5D) | 0.89±0.1 | 0.89±0.1 | 0.83±0.2 | 0.008 (a, b>c) |

a. Subgroup differences were examined by chi-square (categorical) or ANOVA and post-hoc t-tests (continuous).

*Notes: CIRS, Cumulative Illness Rating Scale; DAD, Disability Assessment for Dementia; MCI, mild cognitive impairment; MoCA, Montreal Cognitive Assessment; NCD, neurocognitive disorder; NPI, Neuropsychiatric Inventory; n.s., not significant; SD, standard deviation; VAS: visual analogue scale.*

# Appendix 4. Bivariate correlations between demographics and caregiver outcomes, before and after adjustment

|  | **ZBI** | **PHQ-2** | **Suicidal ideation** | **PACS** |
| --- | --- | --- | --- | --- |
| **(Before adjustment^a^)** | | | | |
| PHQ-2 | .367^***^ | - | - | - |
| Suicidal ideation | .238^***^ | .481^***^ | - | - |
| PACS | .232^***^ | .007 | .024 | - |
| CDR | .560^***^ | .176^***^ | .183^***^ | .331^***^ |
| CR: Gender | .036 | -.135^**^ | -.053 | .096^*^ |
| CR: Age | .410^***^ | .084 | .108^*^ | .309^***^ |
| CR: Education year | -.132^**^ | .029 | .016 | -.144^**^ |
| CR: Married/Cohabited | -.167^***^ | .095^*^ | .004 | -.116^*^ |
| CR: Working | -.126^**^ | -.086 | -.052 | -.086 |
| CR: Household income | -.038 | .020 | .058 | -.044 |
| CR: Cohabitants | .051 | .136^**^ | .090 | -.039 |
| CR: CIRS | .304^***^ | .096^*^ | .007 | .148^**^ |
| CR: MoCA | -.410^***^ | -.116^*^ | -.116^*^ | -.308^***^ |
| CR: DAD | -.508^***^ | -.148^**^ | -.237^***^ | -.288^***^ |
| CR: NPI symptoms | .320^***^ | .170^***^ | .081 | .063 |
| CG: Gender | .163^**^ | .150^**^ | .106^*^ | .042 |
| CG: Age | -.084 | .075 | .082 | -.006 |
| CG: Working | -.028 | -.129^**^ | -.102^*^ | .001 |
| CG: Household income | -.009 | -.079 | .019 | .058 |
| CG: Spouse of CR | -.246^***^ | .066 | .016 | -.158^**^ |
| CG: Cohabit with CR | .007 | .124^**^ | .084 | -.094^*^ |
| CG: Cohabiting children | -.091 | -.074 | -.042 | -.043 |
| CG: Caring hour | .572^***^ | .232^***^ | .194^***^ | .185^***^ |
| CG: Other caregivers | .057 | .030 | -.043 | .098^*^ |
| CG: CIRS | -.048 | .216^***^ | .190^***^ | -.027 |
| CG: Comorbid depression/Anxiety | .135^**^ | .302^***^ | .226^***^ | .082 |
| **(After adjustment^b^)** | | | | |
| PHQ-2 | 0.262^***^ | - | - | - |
| Suicidal ideation | 0.130^**^ | 0.422^***^ | - | - |
| PACS | 0.042 | -0.050 | -0.046 | - |

a. Unadjusted bivariate correlations between different demographics and caregiver outcomes were estimated by Pearson’s correlation coefficient (*, P<0.05, **, P<0.01; ***, P<0.001).

b. Adjusted bivariate correlations among different caregiver outcomes were estimated by partial Pearson’s correlation coefficient, after adjusting for demographics of care-recipient (NCD stage, gender, age, income level, cohabitants, chronic disease, MoCA, DAD, NPI) and caregiver (gender, age, spousal relationship, cohabiting children, caring hours, comorbidity of mood disorders) (*, P<0.05, **, P<0.01; ***, P<0.001).

*Notes: CDR, Clinical Dementia Rating; CG: caregiver; CIRS, Cumulative Illness Rating Scale; CR, care recipient; DAD, Disability Assessment for Dementia; MoCA, Montreal Cognitive Assessment; NPI, Neuropsychiatric Inventory; PACS, Positive aspects of Caregiving scale; PHQ-2, Patient Health Questionnaire-2; SD, standard deviation; ZBI, Zarit Burden Inventory.*

# Appendix 5. Mediation analysis on caring burden, psychological distress and suicidal ideation

|  | Effect | SE | 95%CI | t-value | P-value | Other statistics |
| --- | --- | --- | --- | --- | --- | --- |
| **Step 1. Path estimates** |  |  |  |  |  |  |
| ZBI -> PHQ-2 | 0.038 | 0.008 | (0.022, 0.053) | 4.816 | <0.001 | R^2^=0.211 (P<0.001) |
| PHQ-2 -> SI | 0.098 | 0.012 | (0.075, 0.121) | 8.414 | <0.001 | R^2^=0.215 (P<0.001) |
| ZBI -> SI | 0.001 | 0.002 | (-0.003, 0.005) | 0.463 | 0.643 |  |
| **Step 2. Effect estimates** |  |  |  |  |  |  |
| Direct effect | 0.001 | 0.002 | (-0.003, 0.005) | 0.464 | 0.643 | Mediation: 19.6% |
| Indirect effect | 0.004 | 0.002 | (0.002, 0.007) | 2.467 | 0.023 | Mediation: 80.4% |
| Total effect | 0.005 | 0.002 | (0.001, 0.009) | 2.258 | 0.025 | Mediation: 100% |

Mediation analysis was performed of psychological distress between caring burden and suicidal ideation, controlling for demographics of care-recipient (NCD stage, gender, age, income level, cohabitants, chronic disease, MoCA, DAD, NPI) and caregiver (gender, age, spousal relationship, cohabiting children, caring hours, comorbidity of mood disorders).

*Notes: PHQ-2, Patient Health Questionnaire-2; SI, suicidal ideation; ZBI, Zarit Burden Inventory.*
